# Suicide Trends in Jordan in Correlation With the COVID-19 Pandemic: A Forensic Medicine Perspective

**DOI:** 10.7759/cureus.42636

**Published:** 2023-07-29

**Authors:** Shrouq Al-Sabaileh, Mohammad Abusamak, Hatim Jaber, Ali Al-Buqour, Husam Ahmad AL-Salamat, Radwan Shaher Sabayleh, Hamzeh Hatamleh, Mohammad Ghassab Deameh, Rasmieh M Al-Amer

**Affiliations:** 1 Community, Pathology, and Forensic Medicine, Al-Balqa Applied University, As-Salt, JOR; 2 General and Special Surgery, Al-Balqa Applied University, As-Salt, JOR; 3 Ophthalmology, Amman Eye Clinic, Amman, JOR; 4 Epidemiology and Public Health, Al-Balqa Applied University, As-Salt, JOR; 5 Forensic Medicine, Ministry of Health Al-Hussein/Salt New Hospital, As-Salt, JOR; 6 Internal Medicine, Al-Balqa Applied University, As-Salt, JOR; 7 Internal Medicine, Cairo University, Cairo, EGY; 8 Nursing, Isra University, Amman, JOR

**Keywords:** unemployed, child, pandemic, covid-19 pandemic, self-poisoning, hanging, mental, method, death, suicide death

## Abstract

Background

This study aimed to examine the suicide trends among Jordanians who died by suicide before and during the coronavirus disease 2019 (COVID-19) pandemic and assess their correlation with demographic data, including gender, age, marital status, and the method used for suicide, from a forensic medicine perspective.

Methodology

This was a retrospective, observational, autopsy-based study. Of the 731 medicolegal autopsies performed at the Al-Hussein New Hospital at Al Salt City between 2017 and 2022, 41 confirmed cases of suicide were identified.

Results

The study indicated that 75% of the subjects committed suicide during the COVID-19 pandemic period, of whom 13% were children. The average age of those who died by suicide was 32.1 (SD = 12.2) years. Overall, 58.5% were single, 61.0% unemployed, and 53.7% had a mental illness. Males were four times more likely to commit suicide than females. Hanging represented 60.61% of all male suicides, followed by firearms (27.27%); however, females used poisoning. Around 60% and 25% of the study subjects were in the age group of 21-40 years and 41 and more, respectively. The study also identified a statistically significant association between gender, age, and the time of suicide (during COVID-19). The findings of this study could not confirm any association between suicide and mental illness, marital status, and employment status.

Conclusions

The suicide rate in Jordan increased during the COVID-19 pandemic, and it is alarming that most of those who committed suicide were at a young age and in their productive years.

## Introduction

Suicide is a medicolegal issue that is increasing globally and is a major component of unnatural deaths handled by forensic physicians as part of their routine work. It is estimated that 703,000 people commit suicide globally every year [1]. The risk factors and effects of this public health problem are multifaceted such as poverty, mental illness, genetics, psychological difficulties, and cultural issues [2,3]. For example, in Arabic communities-collectivist nature, “honor killing” is a concept that imposes on females when they have sex out of the marital institution. Sometimes, girls commit suicide because it is almost impossible for them to escape this kind of death [4].

It is widely acknowledged that the coronavirus disease 2019 (COVID-19) has exposed individuals to isolation and fear, specifically during lockdown in which uncertainty was one of the main factors that left individuals in fear for a long time. Hence, individuals felt lonely, depressed, and anxious. Of note, these factors have become more dominant and catastrophic for people around the world [5].

It is worth mentioning that it was observed during the forensic medicine daily casework that the number of suicide cases increased during lockdown worldwide [6,7]; however, this trend was distributed differently across regions and geographical locations. Thus, it is crucial for scholars around the world to add to the body of knowledge related to the distribution of this public health problem by region which is important for global readers and healthcare policymakers.

Hence, it is evident that COVID-19 has a broad and profound impact on a wide range of aspects of individuals’ life and has resulted in an array of mental health difficulties across all countries and among all age groups [8,9]. Of note, many studies reported a strong correlation between COVID-19 and rising suicide rates [2,3,5,7]; nonetheless, not every nation around the world endured similar trends in the number of suicides in response to the pandemic [7,8,10]. In Jordan, during the COVID-19 pandemic, suicide rates have risen to unprecedented levels. According to the most recent Ministry of Statistics estimate, there were 169 suicides in Jordan in 2021, a 10% rise over the previous year and the highest since 2017 [11]. Despite the fact that the increasing number of cases prompted Jordan’s parliament to approve an amendment to the country’s penal code that would punish attempted suicide in public places with up to six months in prison and a fine of 100 Jordanian dinars ($140), the situation in Jordan remains ambiguous due to a lack of sufficient data-driven official reports and accurate statistics on suicide cases [12]. In any event, it is obvious that the COVID-19 epidemic has exacerbated pre-existing life hardships and appears to be partially responsible for a spike in suicide incidents [13].

Although the suicide rate in Jordan has risen during the COVID-19 pandemic, there is a lack of studies investigating the relationship between the suicide rate and the pandemic in Jordan. This autopsy-based observational study aimed to report on the suicide rate before and during the pandemic. Hence, it examined the suicide trends among Jordanians who died by suicide before and during the COVID-19 pandemic and assessed their correlation with their demographic data, including gender, age, marital status, and the method used for suicide from a forensic medicine perspective.

## Materials and methods

Study design, aim, and setting

The aim of this study was to investigate the trends in suicides in the Al-Balqa governorate and report on their correlates from the perspective of forensic medicine. This study was a retrospective, observational, autopsy-based study. The Al-Hussein New Hospital in As-Salt City was the location of this study, which was conducted between 2017 and 2022. A total of 731 medicolegal autopsies were performed at the forensic medicine department of the Al-Hussein government hospital in the governorate of Al-Balqa. The hospital records for these autopsies were reviewed. The overall number of incidents included 41 cases of suicide. The autopsy reports, medical files, and police investigator reports for each case were used to collect data regarding the methods by which the suicide was committed, the mental or psychiatric status, and the socioeconomic characteristics of the deceased. The presence of suicide notes, a prior attempt, the investigation results of the examination at the scene of death, the findings of the autopsy, and the reports of the toxicological examination corroborated the method of suicide. This research was approved by the Ministry of Health’s ethics committee.

Operational definitions

Suicide was defined as “death arising from an act inflicted upon oneself with the intent to kill oneself” [14].In the current study, before COVID-19 is defined as 2017-2019 as the pre-COVID-19 time. During COVID-19 is defined as the years between 2020 and 2022 as the COVID-19 period.

Statistical analysis

For the purpose of the statistical analysis, SPSS (version 29.0.0.1 for Mac) (IBM Corp., Armonk, NY, USA) was utilized. The initial data was gathered by hand and recorded into Excel sheets by the authors, who then performed a second check to ensure that the data was accurate. The de-identified and coded data were imported into SPSS for analysis. The results of the research were divided into two main groups, namely, pre-COVID-19 and during COVID-19. When dealing with continuous variables, we reported the mean as well as the standard deviation, and when dealing with categorical variables, we presented tables of percentage and frequency. In addition, tables of contingency structured in a two-by-two matrix were utilized to investigate the correlation between binary variables utilizing both Pearson’s chi-square and chi-square tests for tables comprising multiple rows.

## Results

Descriptive statistics

The study sample consisted of 41 suicide cases, with an average age of 32.1 (SD = 12.2) years. Age ranged from 8 to 60 years of age, with 80% being males. Overall, 58.5% were single, 61.0% were unemployed, and 53.7% reported having a mental illness. However, the age group of 21-40 years represented 58.5%, followed by the age group of 41+ at 24.4%. Further, 75% of suicide cases were reported during the COVID-19 period. More details are presented in Table 1.

**Table 1:** Demographics characteristics of the study sample (n = 41).

Variables		n	%
Gender	Male	33	80.5
	Female	8	19.5
Age groups (years)	<20	7	17.1
	21–40	24	58.5
	41+	10	24.4
Marital status	Unmarried	24	58.5
	Married	17	41.5
Employment	Unemployed	25	61.0
	Employed	16	39.0
Mental illness	Not declared	19	46.3
	Declared	22	53.7
Pre to during COVID-19	Pre-COVID-19	10	24.4
	During COVID-19	31	75.6

The distribution of cases by age group is depicted in Table 2. In 2017 and 2018, the age distribution of cases was very similar. In 2019, cases were dispersed equitably between the age groups of 21-30 and 41-50 years. In 2020, cases were distributed almost equally among all age groups. In 2022, however, the majority of cases (53.8%) occurred between the ages of 31 and 40, with only one case (7.7%) occurring in those younger than 20 years old.

**Table 2:** Distribution of cases according to the age group (n = 41).

Age (years)	2017	2018	2019	2020	2021	2022
<20	1 (25%)	0 (0%)	0 (0%)	1 (16.6%)	0 (0%)	2 (13%)
21–30	2 (50%)	1 (25%)	1 (50%)	1 (16.6%)	5 (55.5%)	5 (31%)
31–40	0 (0%)	2 (50%)	0 (0%)	2 (33.3%)	2 (22.2%)	7 (44%)
41–60	1 (25%)	1 (25%)	1 (50%)	2 (33.3%)	2 (22.2%)	2 (13%)

Inferential statistics

Table 3 demonstrates the results of Pearson’s chi-square tests for different variables, including gender, age, mental illness, marital status, employment status, and method of suicide comparing the pre-COVID-19 era to the COVID-19 era. The analysis revealed a statistically significant relationship between gender and suicide method (χ^2^ = 15.244, df = 1, p < 0.001), age and suicide method (χ^2^ = 12.050, df = 2, p = 0.002), and pre- to during COVID-19 status and suicide (χ^2^ = 10.760, df = 1, p = 0.001). However, there was no statistically significant association between mental illness, marital status, and employment status with suicide (p > 0.05).

**Table 3:** Test statistics of Pearson’s chi-square for different variables. *: indicates a significant association.

Variable	Pearson’s chi-square test (value, df)	P-value
Gender	15.244^a^, df = 1	<0.001*
Age	12.050^b^, df = 2	0.002*
Mental illness	0.220^a^, df = 1	0.639
Marital status	1.195^a^, df = 1	0.274
Employment	1.976^a^, df = 1	0.16
Method of suicide	33.020^c^, df = 4	<0.001*
Pre-COVID-19 to during COVID-19	10.760^a^, df = 1	0.001*

Method of suicide

Table 4 represents the distribution of suicide methods by gender for the 41 cases. Hanging was the most common method of suicide among males, accounting for 60.61% of all male suicides, followed by firearms (27.27%). However, poisoning was the most common method of suicide for females, accounting for 37.5% of cases. Overall, hanging was the most common method of suicide, accounting for 53.66% of all cases.

**Table 4:** Method of suicide by gender.

Method of suicide	Gender n (%)		Total
	Male	Female	
Falling down	3 (9.09)	2 (25)	5 (12.5)
Firearm	9 (27.27)	0	9 (21.95)
Hanging	20 (60.61)	2 (25)	22 (53.66)
Poison	1 (3.03)	3 (37.5)	4 (9.76)
Stabbing	0	1 (12.5)	1 (2.44)
Total	33 (100)	8 (100)	41 (100)

## Discussion

In the absence of any previous study or official analytical reports on the problem of suicide trends in Jordan during COVID-19, this study aimed to analyze the suicide trends before and during the pandemic, using Al-Balqa, which has the fourth largest population of Jordan’s 12 governorates, with an estimated population of 582,100 at the end of 2022, drawing on cases from a large tertiary center of the rest of the country [15]. The sample was composed of Jordanians who died by suicide.

The study findings indicated that there was a significant increase in the number of suicide cases before and during COVID-19. To illustrate, in Jordan, the percentage of suicide cases has increased by three times in comparison to the period before COVID-19. The study findings supported the previous literature, which reported that during COVID-19 the suicide rate surged worldwide [3,6,10,16]. A potential explanation is that COVID-19 has socially isolated individuals, which, in turn, results in loneliness; the latter represents the subject dimension of an individual’s status, whereas the former refers to the objective dimension of an individual’s life. However, both dimensions are intertwined and impose negative consequences on physical and mental health. Loneliness and social isolation could be prevalent in individualistic communities, not collectivist communities such as Arabs [17]. In Arabic communities, social life is a major determinant of the Arabic cultural norm; hence, it is expected that the lockdown left people feeling lonely, uncertain, and culturally perplexed [18]. Besides, COVID-19 resulted in massive economic drawbacks which increased the levels of economic pressure, particularly on vulnerable women, who could hardly withstand any additional impoverishment, increasing rates of domestic violence [19], and job cuts [20,21]. Needless to say that COVID-19 has been linked to mental health repercussions [22,23]. In this sense, it is important to emphasize the fact that the emergence of female suicide cases during the pandemic is noteworthy. In 2020, there were two incidents, which increased to four by the end of 2022 compared to only one incident in 2017.

The current study revealed that males constituted the majority of suicide cases reported in our center. The study also found a statistically significant relationship between gender and age and the time period before to during COVID-19 status and suicide, respectively. However, there was no statistically significant association between mental illness, marital status, and employment status with suicide. Hanging was the most common method of suicide among males, accounting for 60.61% of all male suicides, followed by firearms (27.27%). Poisoning was the most common method of suicide for females, accounting for 37.5% of cases. Overall, hanging was the most common method of suicide, accounting for 53.66% of all cases.

The age distribution of cases showed no significant change from 2017 to 2022, with the exception of three cases under the age of 18: an eight-year-old boy in 2021, a 15-year-old boy in 2022, and a 12-year-old boy in 2022. The increase in deaths in this age group during the pandemic can be explained in part by isolation and loneliness. However, a wide range of studies has reported video games as risk factors for suicide during COVID-19 [24-26].

The main age groups that showed a predominance were those aged 21-40 years. A possible explanation is that the economic pressure, lower levels of employment rate, social isolation, and loneliness left individuals in despair. According to the most recent World Bank data, the unemployment rate was 18.27% in 2018, which was expected to rise to 19.03% in 2020 [27]. These figures corroborate the findings of our study, which found that the number of suicides among unemployed persons increased between 2020 and 2022 compared to 2018 and 2019 [28].

Although some epidemiological studies have reported that marital status correlates with suicide, our study did not find any correlation. This may be partly attributed to the fact that males and females equally suffer from the consequences of COVID-19.

The preferred methods of suicide among the cases in our study were hanging and firearms, which corresponds to global trends toward these methods of suicide [7,29]. It is worth noting that poisoning by pesticide was the favored method for suicide among female cases in this study while hanging was the preferred means of suicide among those under the age of [20,30,31].

The correlation between mental or psychiatric disorders and suicide is well-established, with such disorders often serving as a major risk factor or even a direct cause [22,23]. However, in Jordan, we could not find a trace of any correlation, which could be explained by the fact in collectivist communities, the social stigma surrounding suicide events makes it difficult for forensic physicians to obtain reliable data regarding the true mental or psychiatric status of deceased individuals.

Study limitations

This study had a few limitations, including a small sample size, and the authors were unable to verify the mental state of some of the subjects in some of the records examined. This is due in part to the loss of contact methods with the families and the refusal of some families to address their son’s or daughter’s suicide owing to social taboos and shame. Both of these factors contribute to this issue.

## Conclusions

This study showed a significant increase in the rates due to the negative impact of the COVID-19 pandemic. The emergence of suicide cases among children and the increasing trend among females during the pandemic in comparison to the pre-pandemic period were unprecedented. Therefore, there is an urgent need for a national strategy that involves multiple sectors to create a national analytical database for suicide cases and identify vulnerable subpopulations. The government should prioritize the implementation of protective measures, such as mental and psychological public health services, and efforts to reduce social stigmatization, improve economic status, and create effective policies to reduce unemployment.
